# Notices

**Published:** 1862-11

**Authors:** 


### NOTICES.

THE attention of the medical profession is invited to the patronage of *The Journal of Materia Medica, Pharmacy, Chemistry, etc.*, conducted by Joseph Bates, M.D., and H. A. Tilden, New-Lebanon, N. Y. Published monthly at one dollar a year. We have for some time past derived most valuable hints in materia medica, therepeutics, and practical medicine from this judiciously conducted monthly, now in its fourth volume, new series. Vols. I., II., and III., uniformly bound, are furnished at one dollar each, giving the latest improvements and discoveries throughout the world, as to the nature, uses, and combinations of all medical substances.

MORAL REMEDIES FOR PHYSICAL AND MENTAL DISEASES. By REV. GEO. A. LEAKIN, A.M., designed mainly for the hospital; as a previous tract by the same author was intended principally for the soldier in camp, entitled: *The Soldier in Algeria.*

The topic is one of great practical interest, and it is to be hoped that the reverend author, and others of high culture and mental power, will pursue the subject farther; exemplifying in various ways that there is quite as great therapeutic power in moral remedies as in drugs. The JOURNAL OF HEALTH has insisted upon this to such an extent that the complaint has come to the editor that, if the JOURNAL said less about morals and religion, it would be still more acceptable. The proper object of a journal of health, for popular reading, is to show how health may be preserved, and how life may be prolonged. But if in doing this we were restricted to subjects connected with eating and drinking, with rest and exercise, with sleep and work, with abstinence and temperance, we should be confined to half of our legitimate field; because the practice of the virtues, and the cultivation of the religious sentiments, do certainly tend to prevent disease, to alleviate it, and to cure. Divinity has said, "The wicked shall not live out half their days," leaving us to infer that the righteous will; and if we can induce men and women and children to practice that righteousness, as exemplified in being "temperate in all things," in cultivating "cleanliness," and "fidelity," doing justly, loving mercy, practicing benevolence to the fatherless and the widow, and living unspotted from the world—all of which are religious duties—if we can lead our readers to "follow" these things, we think we shall be guiding them into those paths which lead to length of days on earth and a beatific existence beyond the grave.

NEW-YORK OPHTHALMIC HOSPITAL, 387 Fourth Avenue, incorporated ten years ago, has steadily increased in usefulness under Prof. Stephenson and Dr. Gerrish, both men of eminent practical ability. That noble specimen of great-heartedness, Peter Cooper, is the president, and the veteran Dr. Mott, of world-wide fame, is the consulting surgeon, while such men as Moses H. Grinnell, James Bowman, Chancellor Ferris, and William B. Astor, are among its trustees. Nine thousand three hundred and forty-five patients have been admitted for the treatment of eye diseases; many of whom have gone away entirely cured. Only one hundred and seventy out of the whole number are reported incurable.

SOLDIER HEALTH sent, prepaid, for 5 cents; one dozen sent for 50 cents, prepaid; $2.50 per hundred; $20 per thousand, delivered at 42 Irving Place, New-York.

DR. W. W. HALL, of New-York, editor of the well-known JOURNAL OF HEALTH, has published new editions of his four valuable works upon BRONCHITIS AND KINDRED DISEASES; CONSUMPTION; HEALTH AND DISEASE; and SLEEP. They are filled with sensible, practical advice, given in a comprehensible, fluent style, and naturally treat upon a large variety of topics, among which are consumption, apoplexy, ventilation of rooms, food, lungs, sea-voyages, debilitations, cold feet, flannels, and every thing, in fact, conducing to health and disease, protection, prevention, exercise, attire, etc., etc. A vast deal of research, experience, and care are exhibited in the books, and their tendency is to instruct and benefit in the most direct manner. The laws of nature are explained, the necessity of observing them inculcated, and the evils of irregularity, excess, and abuse vividly presented. There is so much valuable information in these works, and evidently such patient, discriminating labor in their preparation, that a newspaper paragraph fails to render the author justice. But we commend them as useful to every man and woman. Dr. Hall throws light upon certain subjects which are unfortunately too little comprehended—matters obviously not to be dwelt upon here, and what he says is delicately and sagaciously told. He warns the public against a certain class of publications on physiology as pernicious, giving conclusive reasons for his opinion.* The four books are well printed and neatly bound, and may be obtained of the Doctor at a price which, considering their intrinsic value, is indeed moderate.—*Boston Post.*

* IN SLEEP sent, post prepaid, for $1.40, and the others for $1.15 each.

